# The research on infrared radiation affected by smoke or fog in different environmental temperatures

**DOI:** 10.1038/s41598-024-65462-x

**Published:** 2024-06-22

**Authors:** Huaizhou Li, Shupei Wen, Sen Li, Hong Wang, Xin Geng, Shuaijun Wang, Jinlong Zhai, Wenhua Zhang

**Affiliations:** College of Building Environmental Engineering, Zhengzhou University of Light Industry, Zhengzhou, 450001 China

**Keywords:** Infrared thermal imaging, Fog environment, Smoke environment, Optics and photonics, Physics

## Abstract

Infrared thermal imaging camera as a non-contact monitoring of the object to be measured is widely used in fire detection, driving assistance and so on. Although there are many related studies, there is a lack of research on the influence of fog or smoke on infrared imaging under different environmental temperatures. To address this shortcoming, The temperature of both the environment and the target in this experiment is controlled by PID technology. The smoke or fog environment is generated using a smoke cake or an ultrasonic fog machine. The temperature of the target was measured by infrared thermal imaging camera. It was observed that as the temperature of the environment increases, the measured temperature of the target also increases. However, the change in temperature is more pronounced in the fog environment compared to either the smoke environment or the normal environment. It has been found through research that environmental radiation causes temperature changes in fog droplets. Therefore, Infrared radiation is less affected in the smoke environment and more affected in the fog environment. Additionally, when the environmental temperature is close to the target's temperature, the infrared image becomes blurred.

## Introduction

**Figure 1:**
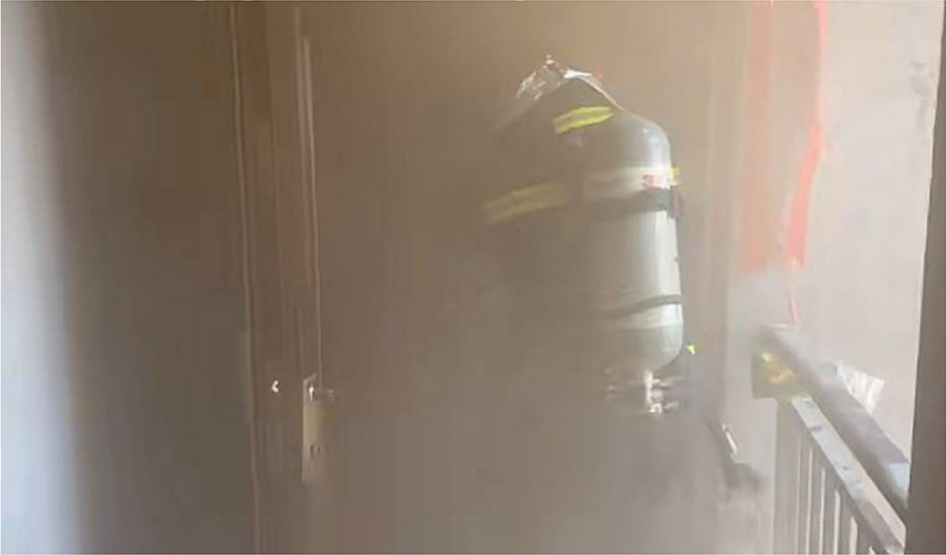
The fire scene (from Fuyang fire protection).

Thermal imaging serves as a non-contact detection method, producing images by capturing infrared radiation emitted from an object's surface^1^. It enables the visualization of target objects under varying environmental temperatures, finding applications in fire detection^2^, power grid risk identification^3^, driving assistance^4^, medicine^5^, and more. For instance, during fire incidents, substantial smoke is produced, rendering visible light penetration impossible, as illustrated in Fig. 1. However, infrared thermography can transcend the limitations of visible light, thereby enabling the visualization of target objects. Nonetheless, smoke also introduces certain interferences with infrared thermography. Consequently, this paper concentrates on examining the impact of infrared thermal imaging in smoke and fog environments.

Numerous studies have explored the effect of infrared thermal imaging in smoke or fog environments. For instance, Ijaz et al. conducted experiments on the attenuation of an FSO communication system in a controlled laboratory smoke environment, comparing the results with a theoretical smoke model ^6^. Gilles Parent et al. investigated the attenuation of infrared radiation through a mixture of spray, smoke, and both media, analyzing the attenuation by mixing^7^. Jaap de Vries et al. measured instantaneous and time-averaged blackbody temperature profiles through a flame using an LWIR microbolometer camera ^8^. Mingxing Zhang et al. evaluated the performance of nine types of smoke curtains, focusing on attenuation and hanging capabilities^9^, while Du Shiming et al. analyzed the extinction mechanism of infrared smoke curtains^10^. Momma et al. conducted a study on the scattering and attenuation phenomena of near-infrared light in particles comprising fog and smoke^11^. Songtao Liu et al. designed a probabilistic model for infrared imaging target detection, offering guidelines and methods for evaluating the effect of smoke interference^12^. Michael P. Thornton et al. quantified the performance of thermal infrared sensors under different fog conditions^13^, and R. Nebuloni et al. analyzed the effect of fog on attenuation at various wavelengths^14^. Kelsey M. Judd et al. presented qualitative performance data for various imaging sensors under fog in different chambers^15^. Ijaz M. et al. compared the performance of a free-space optical communication system in a controlled laboratory fog environment^16^. Ting Wang et al. analyzed the effects of visibility, transmission distance, incident wavelength, and calculated values of clear atmospheric transmittance on the total transmittance of advective and radiative fogs using different infrared radiations^17^. Wei Li et al. conducted imaging experiments in sea fog, quantifying the attenuation model of infrared radiation in sea fog^18^. Lei Lizhi performed calculations of the infrared radiation characteristics in the wavelength band of 3–5 μm in hazy weather^19^, while Li et al. quantitatively analyzed the extinction coefficients of different sea mists based on the scattering and absorption characteristics of infrared radiation in sea mists^20^. Xu Dongxiang et al. analyzed the attenuation capacity of infrared radiation in water mists in different directions^21^. Leonid A. Dombrovsky et al. estimated the effect of scattering of flame infrared radiation by small droplets on the radiation source function^22^.

While there has been considerable research in this field, there is a relative scarcity of studies examining the effects of fog or smoke environments on infrared imaging under varying environmental temperatures. This paper aims to investigate how infrared thermography is influenced by smoke or fog in different environmental temperatures, providing theoretical insights and practical guidance for the application of thermal imaging technology in complex environments.

The experimental setup in this study involves generating smoke environments by burning smoke cakes and creating fog environments using ultrasonic foggers. A direct current fan is used to evenly distribute smoke or fog throughout the experimental platform space. Additionally, electronic components such as microcontrollers and MOS transistors are used to regulate and control the temperature of the fog, target objects, and ambient environment using PID adjustments. Infrared thermal imagers are used to capture both the temperature of target objects and images.This paper is structured into five main sections. The first section provides an overview of current research on infrared thermography. The second section introduces the experimental platform setup. The third section analyzes the theoretical aspects of laser attenuation and thermal imaging attenuation during measurements. The fourth section presents experimental data on infrared thermography in smoke or fog environments at different temperatures, followed by data analysis and comparison. Finally, the fifth section presents the conclusions drawn from the study.

The practical implications of this study are significant. For example, during firefighting operations, heavy smoke and fog are often present, while risk identification in power grids and automotive assisted driving can also be affected by dense fog conditions. Therefore, this study can provide theoretical foundations and practical guidance for the application of infrared thermography in such scenarios.

## Methods

### Construction of the experimental platform

**Figure 2:**
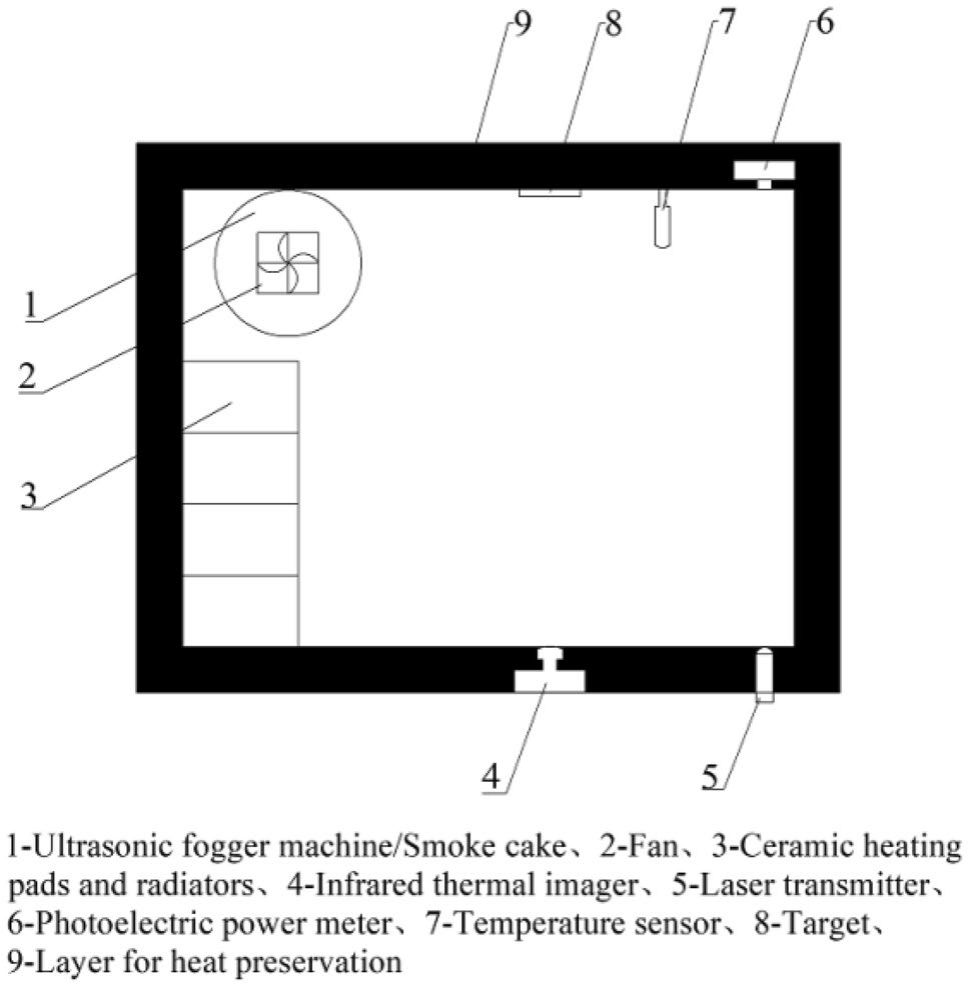
Layout of infrared thermal imaging lab bench.

This experimental platform is designed to investigate the impact of smoke or fog environments on infrared thermal imaging at varying temperatures. The key equipment employed in this experiment includes an ultrasonic fog machine, smoke cakes, a fan, a ceramic heater, a heatsink, an infrared thermal imaging camera, a laser transmitter, a photoelectric power meter, temperature sensors, an MCU, MOS tubes, and other essential components. The layout of the experimental platform is illustrated in Fig. 2.

The structural framework of the entire testing setup is crafted from acrylic boards, creating a confined space measuring 400mm × 300mm × 300mm. This space is enclosed in thermal insulation cotton to shield the internal temperature from external influences. Corresponding apertures and positions are integrated into the outer wall of the experimental platform for tasks such as capturing images, conducting data measurements, and arranging power cords.

**Figure 3:**
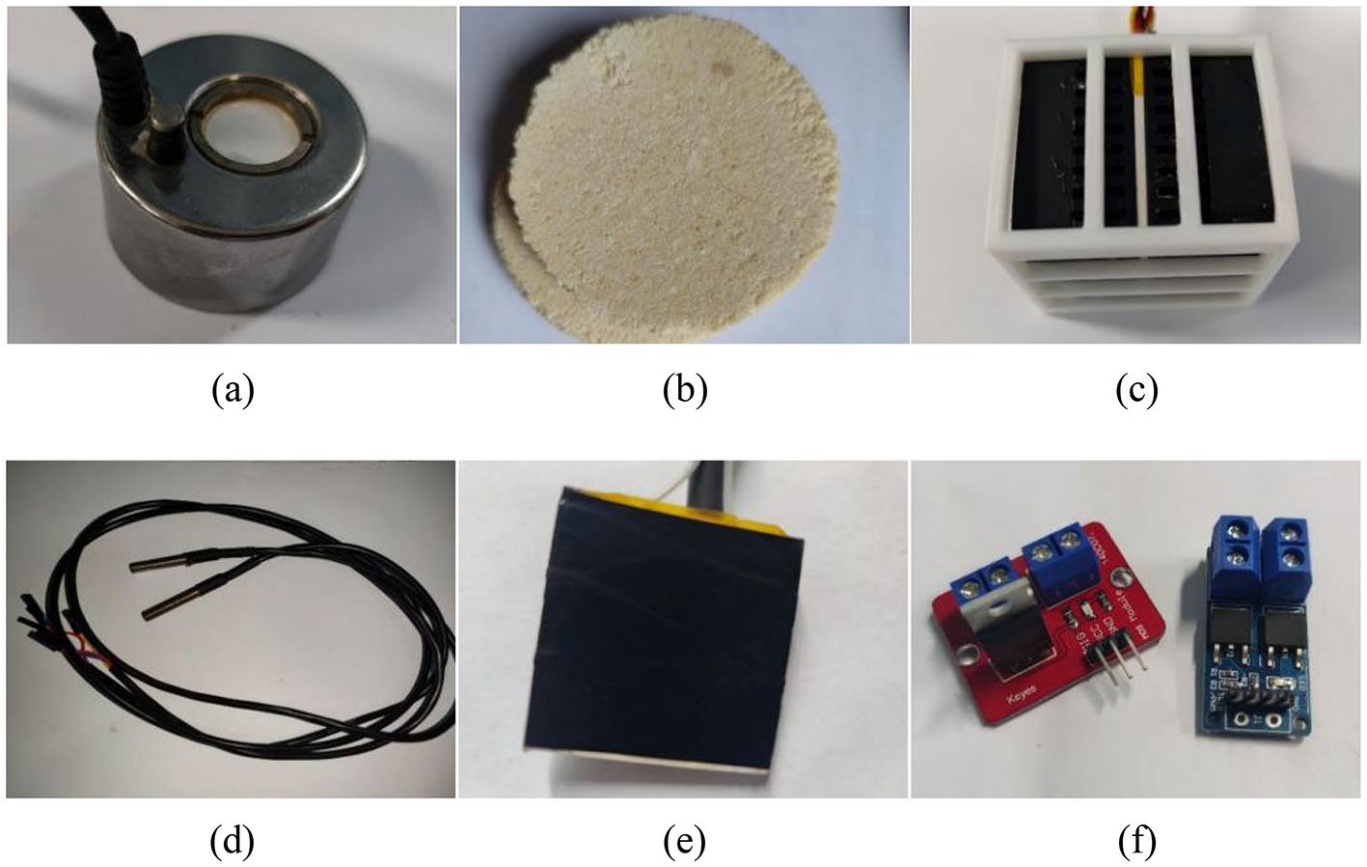
The experimental apparatus. (**a**) Ultrasonic atomizer. (**b**) Smoke cartridges. (**c**) Heating device. (**d**) Temperature sensor. (**e**) Target. (**f**) MOS transistor.

Fog is generated using the ultrasonic fog machine and dispersed throughout the entire experimental platform by the fan. The size and consistency of the fog are meticulously controlled by evaluating the attenuation of the 405nm laser emitted from the laser transmitter. This evaluation is conducted through the optoelectronic power meter, ensuring the stability and uniformity of the fog across the entire testing platform. Figures 3a depict the ultrasonic fog machine.

Smoke is generated by igniting the smoke cake and then propelled throughout the space by a fan. The visibility of the smoke is quantified using an optoelectronic power meter equipped with a laser emitter. Figure 3b provides a visual representation of the smoke cake used in the experiment.

The heating apparatus comprises four sets of ceramic heating pads, heat sinks, and fans. Utilizing fans and heat sinks, the heat generated from the ceramic heating pads is evenly distributed throughout the interior of the experimental platform. Temperature sensors are strategically positioned near the target, and their data are employed to regulate the heating device, ensuring the stability of the temperature within the experimental platform. Figure 3c depicts the heating device, while Fig. 3d illustrates the temperature sensor.

Due to the elevated external environmental temperature, which fails to meet the experiment's low-temperature requirements, the internal environmental temperature within the experimental platform is meticulously regulated. This is achieved by incorporating a DC small fan and ice. The cold air generated by the ice is disseminated throughout the entire experimental platform via the fan. Simultaneously, the temperature inside the platform is closely monitored by temperature sensors, influencing the rotational speed of the fan to maintain temperature stability.

The target consists of a ceramic heating pad and a temperature sensor. To secure both components together, black electrical insulating tape is employed. The temperature sensor governs the heating power of the ceramic heating pad, ensuring the constancy of the target temperature. Figure 3e is the target.

To guarantee the stability of the target temperature within the experimental platform, a STC89C516R + microcontroller and MOS tubes are employed. These components utilize an incremental PID algorithm for precise control. The control equation for the PID algorithm ^23^ is illustrated in Eq. (1). The image of the MOS transistor is shown in Fig. 3f.

1 $$ \left\{ {\begin{array}{*{20}l} {\Delta {\text{u}}_{{\text{k}}} {\text{ = K}}_{{\text{p}}} \times \left( {{\text{e}} - {\text{e}}_{1} } \right){\text{ + K}}_{{\text{i}}} \times {\text{e + K}}_{{\text{d}}} \times \left( {{\text{e}} - {2} \times {\text{e}}_{{1}} {\text{ + e}}_{{2}} } \right)} \hfill \\ {{\text{u}}_{{\text{k}}} { = }\Delta {\text{u}}_{{\text{k}}} {\text{ + u}}_{{{\text{k1}}}} } \hfill \\ \end{array} } \right. $$

where ${\text{e}}$ is the current deviation value variable,${\text{e}}_{1}$ is the subsequent deviation value variable,${\text{e}}_{2}$ is the further deviation value variable,${\text{K}}_{\text{p}}$ is the proportionality coefficient,${\text{K}}_{\text{i}}$ is the integration coefficient,${\text{K}}_{\text{d}}$ is the differentiation coefficient,${\text{u}}_{\text{k}}$ is the total current deviation value variable,${\text{u}}_{\text{k}1}$ is the total subsequent deviation value variable and ${\Delta \text{u}}_{\text{k}}$ is the total deviation value variable.

**Figure 4:**
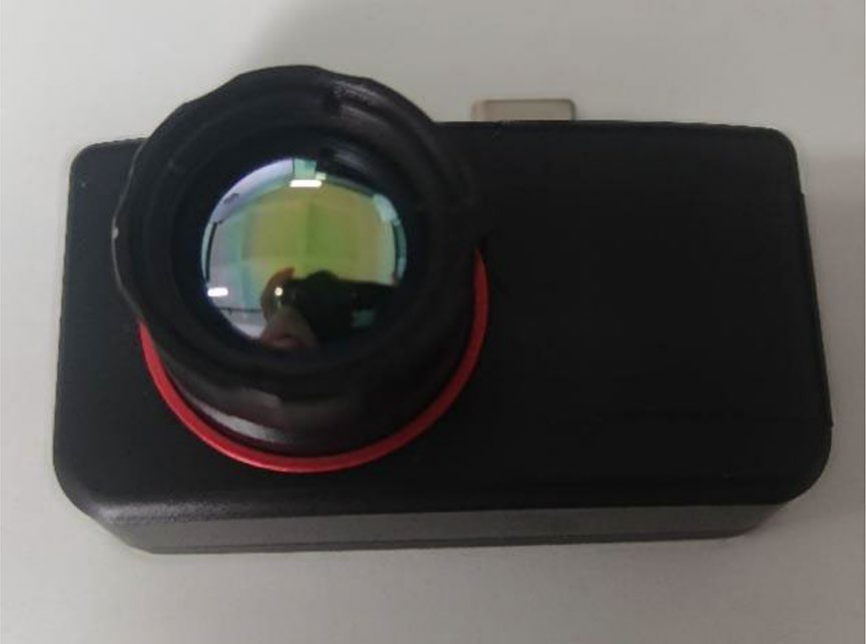
The Infrared thermal imaging camera.

This infrared thermal imaging camera is sensitive to wavelengths within the range of 8-14μm and has a temperature detection span from − 20 to + 400 °C. Within this range, the camera's error is ± 2 °C. Since the measured temperatures primarily fall between 20 °C and 50 °C, the infrared thermal imaging camera 's operational range is chosen to be − 20 to 120 °C. Within this range, the camera's error is ± 0.667 °C. The field of view angle of this infrared thermal imaging camera is 30 ° x 22.5 ° Notably, the infrared thermal imaging camera allows for the correction of deviations directly at its terminals. The image of the infrared thermal imaging camera is shown in Fig. 4.

### The theoretical analysis of experiments

#### The theoretical analysis of the visibility of smoke or fog

The attenuation of the laser's intensity ${\text{I}}_{0}$ after transmission over a distance L mainly depends on the transmittance of the laser beam T^24–26^, as described in Eq. (2).

2 $$  {\text{T = I/I}}_{{\text{0}}} {\text{ = exp}}\left( { - \int\limits_{{\text{0}}}^{{\text{L}}} {\upbeta {\text{dl}}} } \right)  $$

where I is the light intensity after the transmission distance L, and $\upbeta$ is the atmospheric attenuation coefficient.

The ultrasonic fog machine produces fog particles with an average diameter of 0.4μm ^27^, while the combustion of the smoke cake yields smoke particles with an average diameter of 160 nm ^26^. Given that the laser's wavelength is 405 nm, the Mie scattering principle is applicable. The attenuation formula for laser light is expressed in Eqs. (3) and (4) ^28^.

3 $$  \upbeta {\text{ = (3}}{\text{.912/V)}} \times {\text{(0}}{\text{.55/}}\uplambda {\text{)}}^{{\text{q}}}   $$

4 $$ {\text{q = }}\left\{ {\begin{array}{*{20}l} {0} \hfill & {{\text{(D < 0}}{.5}\;{\text{km)}}} \hfill \\ {{\text{D}} - 0.5} \hfill & {{(0}{\text{.5}}\;{\text{km < D < 1}}\;{\text{km)}}} \hfill \\ {{0}{\text{.16D}}\;{ + }\;{ 0}{\text{.34}}} \hfill & {{(1}\;{\text{km < D < 6}}\;{\text{km)}}} \hfill \\ {{1}{\text{.3}}} \hfill & {{(6}\;{\text{km < D < 50}}\;{\text{km)}}} \hfill \\ {1.6} \hfill & {{\text{(D > 50}}\;{\text{km)}}} \hfill \\ \end{array} } \right. $$

where V is the meteorological visibility,$\uplambda$ is the wavelength of the laser, D is the measurement distance.

After the collation of formula (2) (4), the meteorological visibility formula is obtained as Eq. (5).

5 $$ {\text{V = }}\frac{ - 3.912}{{{\text{ln}}\frac{{\text{I}}}{{{\text{I}}_{{0}} }}}}\left( {\frac{{{0}{\text{.55}}}}{{\uplambda }}} \right)^{{\text{q}}} {\text{L}} $$

#### The theoretical analysis of infrared thermography affected by smoke or fog

Infrared thermal imaging is influenced by atmospheric conditions, wherein the infrared radiation emitted by the target undergoes alterations as it interacts with smoke or fog before reaching the infrared thermal imaging camera. This process is intricate, involving the absorption and scattering of infrared radiation. Relevant theories such as the Lambert–Beer law, Mie scattering, Rayleigh scattering, and others are integral components in understanding this phenomenon.

##### The theoretical analysis of attenuation in normal and fog environment

The infrared thermal imaging temperature measurement calculation formula is shown in Eq. (6) ^29^.

6 $$  {\text{T}}_{{\text{0}}} {\text{ = }}\left\{ {\frac{{\text{1}}}{\upvarepsilon }\left[ {\frac{{\text{1}}}{{\uptau _{{\text{a}}} }}{\text{T}}_{{\text{r}}}^{{\text{n}}}  - {\text{(1}} - \upvarepsilon {\text{)T}}_{{\text{u}}}^{{\text{n}}}  - \left( {\frac{{\text{1}}}{{\uptau _{{\text{a}}} }} - {\text{1}}} \right){\text{T}}_{{\text{a}}}^{{\text{n}}} } \right]} \right\}^{{{\text{1/n}}}}   $$

where ${\text{T}}_{0}$ is the true temperature of the object to be measured, ε is the emissivity of the object to be measured, ${\uptau }_{{\text{a}}}$ is the atmospheric transmittance,${\text{T}}_{{\text{r}}}$ is the temperature measured by the infrared thermal imaging, ${\text{T}}_{{\text{u}}}$ is the environmental temperature, ${\text{T}}_{{\text{a}}}$ is the atmospheric temperature, and n is a constant related to the wavelength of the infrared thermal imaging. n is 9.2554 when the wavelength is 3–5 μm, and n is 3.9889 when the wavelength is 8–12 μm ^30^.

Atmospheric attenuation of infrared radiation is mainly related to four phenomena; (1) absorption of water vapour; (2) absorption of carbon dioxide; (3) scattering of molecules, aerosols and particles in the atmosphere; (4) attenuation of meteorological conditions. Therefore, the transmittance of infrared radiation to the atmosphere is also composed of four parts, as shown in Eq. (7).

7 $$  \uptau _{{\text{a}}} {\text{ = }}\uptau _{{{\text{H}}_{{\text{2}}} {\text{O}}}}  \times \uptau _{{{\text{CO}}_{{\text{2}}} }}  \times \uptau _{{\text{p}}}  \times \uptau _{{\text{R}}}   $$

where ${\uptau }_{{{\text{H}}_{{2}} {\text{O}}}}$ is the infrared radiation transmittance after absorption by water vapour, ${\uptau }_{{{\text{CO}}_{{2}} }}$ is the infrared radiation transmittance after absorption by carbon dioxide, ${\uptau }_{{\text{p}}}$ is the infrared radiation transmittance after scattering, and ${\uptau }_{{\text{R}}}$ is the infrared radiation transmittance after attenuation by rain, snow and so on.

In the experiment, there is no rain, snow and other weather phenomena, so this experiment don’t consider ${\uptau }_{{\text{R}}}$.

(1) Calculation of water vapour attenuation.

The determination of ${\uptau }_{{{\text{H}}_{{2}} {\text{O}}}}$ needs to refer to the concept of "the content of water vapour after a certain distance of transmission in the sea level ${\upomega }$", the value of ${\upomega }$ is calculated according to the formula (8).

8 $$   \upomega {\text{  =  H}}_{{\text{r}}}  \times {\text{H}}_{{\text{a}}}  \times {\text{D}} $$

where ${\text{H}}_{\text{a}}$ represents the content of saturated water vapor, The relative humidity ${\text{H}}_{\text{r}}$ is 27% in normal conditions, 100% in foggy environments, and the transmission distance D is 0.3 m.

Based on the calculated value of ω and the detection band of the infrared thermal imaging, the water vapour transmission rate for the band can be found in the relevant sources ^31^.

(2) Calculation of carbon dioxide attenuation.

According to relevant studies, the density of carbon dioxide remains practically constant in the atmosphere in the surface layer. Therefore ${\uptau }_{{{\text{CO}}_{{2}} }}$ is related to the distance through which the radiation passes, and the value of ${\uptau }_{{{\text{CO}}_{{2}} }}$ is also given in the relevant sources, which can be consulted directly ^32^.

(3) Calculation of scattering by molecules and particles in the atmosphere.

In the process of infrared radiation, it will be refracted by molecules and particles in the atmosphere, etc. The attenuation coefficient of the infrared radiation after attenuation by atmospheric molecules and particles can be calculated according to Eq. (4). From the attenuation coefficient can be based on the Lambert–Beer law to derive the corresponding wavelength of the atmospheric transmittance as shown in Eq. (9).

9 $$  \uptau _{{\text{P}}} {\text{  =  exp(}} - \beta {\text{D}}) $$

where $\upbeta$ is the attenuation factor, which can be obtained by formula (4) , the distance D between the target object and the infrared thermal imager is 0.3 m, and e is a constant.

##### The theoretical analysis of attenuation in smoke environment

In smoke environment, the atmospheric transmittance of infrared radiation is mainly composed of two components, absorption and scattering, and the scattering rate is shown in Eq. (10) ^33^.

10 $$  \uptau _{{\text{a}}} {\text{  =  }}\uptau _{\alpha }  \times {\text{ }}\uptau _{\beta }     $$

The attenuation due to absorption obeys Beer's law as shown in Eq. (11).

11 $$ {\uptau }_{{\upalpha }} {\text{ = exp(}} - {\upsigma }_{{\text{a}}} {\text{cD)}} $$

where D represents the distance from the infrared thermal imager to the target object, the absorption extinction coefficient ${\upsigma }_{{\text{a}}}$ of smoke is ${1}{\text{.5}}\;{\text{g/m}}^{{2}}$, and the mass concentration c of smoke is ${0}{\text{.885}}\;{\text{g/m}}^{{3}}$.The attenuation due to scattering is shown in Eq. (12).

12 $$ {\uptau }_{{\upbeta }} {\text{ = exp(}} - {\text{k}}_{{\text{s}}} {\text{ND)}} $$

where ${\text{k}}_{\text{s}}$ is the scattering attenuation cross section, which can be obtained from the following Eq. (13).

13 $$ {\text{k}}_{{\text{s}}} {\text{ = N}}\uppi {\text{r}}^{{\text{2}}} {\text{Q}}_{{{\text{sca}}}}   $$

where D represents the distance from the infrared thermal imager to the target object, the number density N of scattering particles in smoke is ${5}{\text{.9}} \times {10}^{{{14}}} { }\;{\text{particles/m}}^{{3}}$, the radius r of smoke particles is 180 nm, and ${\text{Q}}_{\text{sca}}$ is the scattering efficiency factor.

To describe the problem intuitively, a size factor x is introduced as shown in Eq. (14).

14 $$  {\text{x = 2}}\uppi {\text{r/}}\uplambda   $$

where the wavelength λ of the incident light is 8–14 µm.

When the size of the smoke screen particles is small, usually referred to as x < 0.3, the scattering obeys Rayleigh's law ^34^. It is known from the electromagnetic field theory of light that the scattering intensity currently is inversely proportional to the fourth power of the wavelength of the incident laser. The scattering efficiency factor is shown in Eq. (15).

15 $$  {\text{Q}}_{{{\text{sca}}}} {\text{ = }}\frac{{{\text{128}}\uppi ^{{\text{4}}} {\text{r}}^{{\text{4}}} }}{{{\text{3}}\uplambda ^{{\text{4}}} }}\left( {\frac{{{\text{m}}^{{\text{2}}}  - {\text{1}}}}{{{\text{m}}^{{\text{2}}} {\text{ + 1}}}}} \right)^{{\text{2}}}  $$

If expressed in terms of the size parameter x, it is shown in Eq. (16).

16 $$ {\text{Q}}_{{{\text{sca}}}} { = }\frac{{8}}{{3}}{\text{x}}^{{4}} \left( {\frac{{{\text{m}}^{{2}} - {1}}}{{{\text{m}}^{{2}} { + 1}}}} \right)^{{2}} $$

where the refractive index m of smoke particles is 1.4.

## Results

### Collection of experimental data

**Figure 5:**
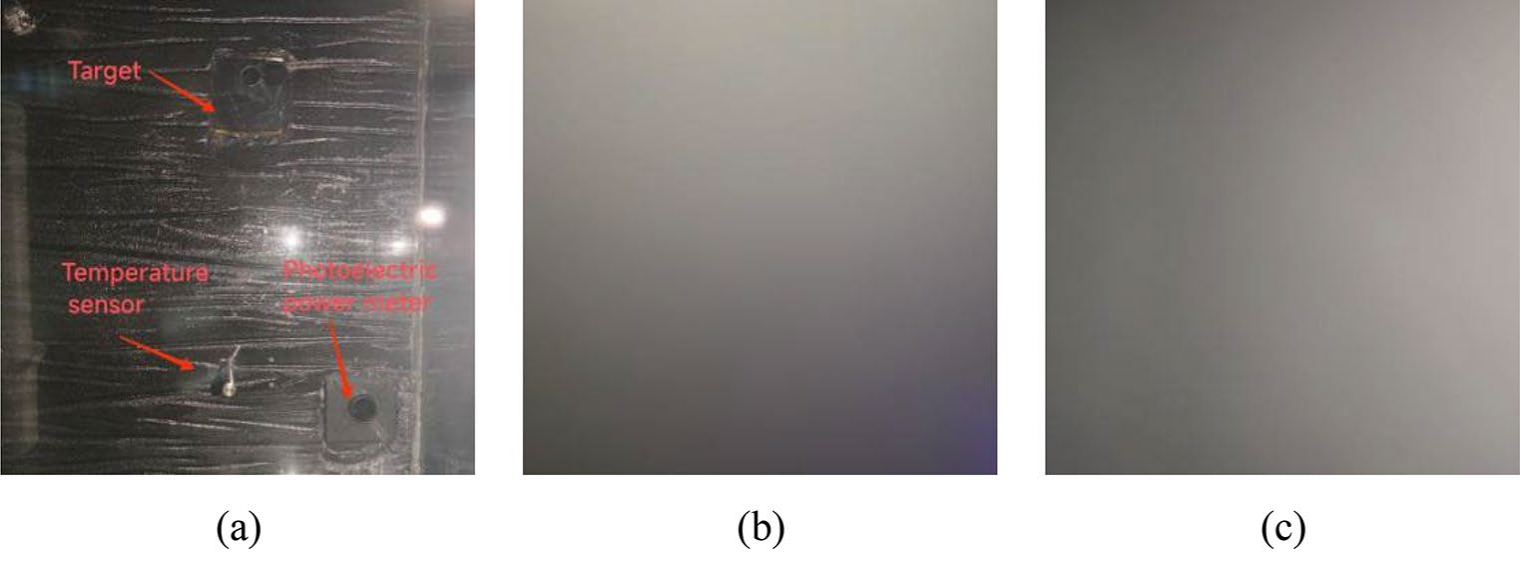
Images taken by the visible camera in normal, smoke and foggy environments. (**a**) Normal environment. (**b**) Smoke environment. (**c**) In a foggy environment.

Images of the target captured by the visible camera in normal, smoke, and fog environments are presented in Fig. 5.

The initial light intensity of the 405 nm laser transmitter, with a power of 1 W, measured by the photoelectric power meter under normal conditions, is 701.812 $\text{mW/}{\text{cm}}^{2}$. The light intensity ($\text{mW/}{\text{cm}}^{2}$) of the laser transmitter measured by the photoelectric power meter after attenuation is presented in Table 1 under different environmental temperatures in the smoke environment. Table 2 displays the light intensity ($\text{mW/}{\text{cm}}^{2}$) measured by the photoelectric power meter after attenuation of the laser transmitter in the fog environment at various environmental temperatures. To ensure data accuracy, ten sets of measurements were taken at different environmental temperatures in smoke or fog environments, and the average values were used for meteorological visibility calculations.

**Table 1:** The light intensity of a laser transmitter after passing smoke at different environmental temperatures.

Environmental temperature	Attenuated light intensity ($\text{mW/}{\text{cm}}^{2}$)										
	1	2	3	4	5	6	7	8	9	10	Mean
20 °C	1.23	1.25	1.21	1.19	1.23	1.24	1.23	1.26	1.22	1.23	1.229
25 °C	1.21	1.23	1.19	1.21	1.24	1.22	1.19	1.21	1.21	1.23	1.214
30 °C	1.22	1.21	1.24	1.23	1.28	1.21	1.25	1.24	1.23	1.21	1.232
35 °C	1.25	1.18	1.23	1.23	1.23	1.25	1.19	1.21	1.23	1.22	1.222
40 °C	1.20	1.21	1.26	1.25	1.22	1.21	1.25	1.23	1.21	1.19	1.223
45 °C	1.25	1.22	1.23	1.18	1.21	1.25	1.23	1.23	1.22	1.21	1.223
50 °C	1.19	1.21	1.28	1.27	1.22	1.23	1.22	1.25	1.20	1.22	1.229

**Table 2:** The light intensity of a laser transmitter after passing through fog at different environmental temperatures.

environmental temperature	Attenuated light intensity ($\text{mW/}{\text{cm}}^{2}$)										
	1	2	3	4	5	6	7	8	9	10	Mean
20 °C	2.22	2.22	2.22	2.23	2.22	2.26	2.25	2.22	2.22	2.22	2.228
25 °C	2.23	2.22	2.24	2.22	2.23	2.23	2.23	2.22	2.24	2.23	2.229
30 °C	2.22	2.22	2.23	2.23	2.22	2.21	2.22	2.23	2.21	2.21	2.220
35 °C	2.18	2.21	2.19	2.18	2.19	2.22	2.21	2.20	2.17	2.21	2.196
40 °C	2.25	2.22	2.27	2.26	2.22	2.22	2.28	2.22	2.22	2.21	2.237
45 °C	2.22	2.28	2.25	2.23	2.20	2.19	2.23	2.23	2.23	2.29	2.235
50 °C	2.27	2.22	2.21	2.25	2.22	2.22	2.21	2.19	2.22	2.22	2.223

**Figure 6:**
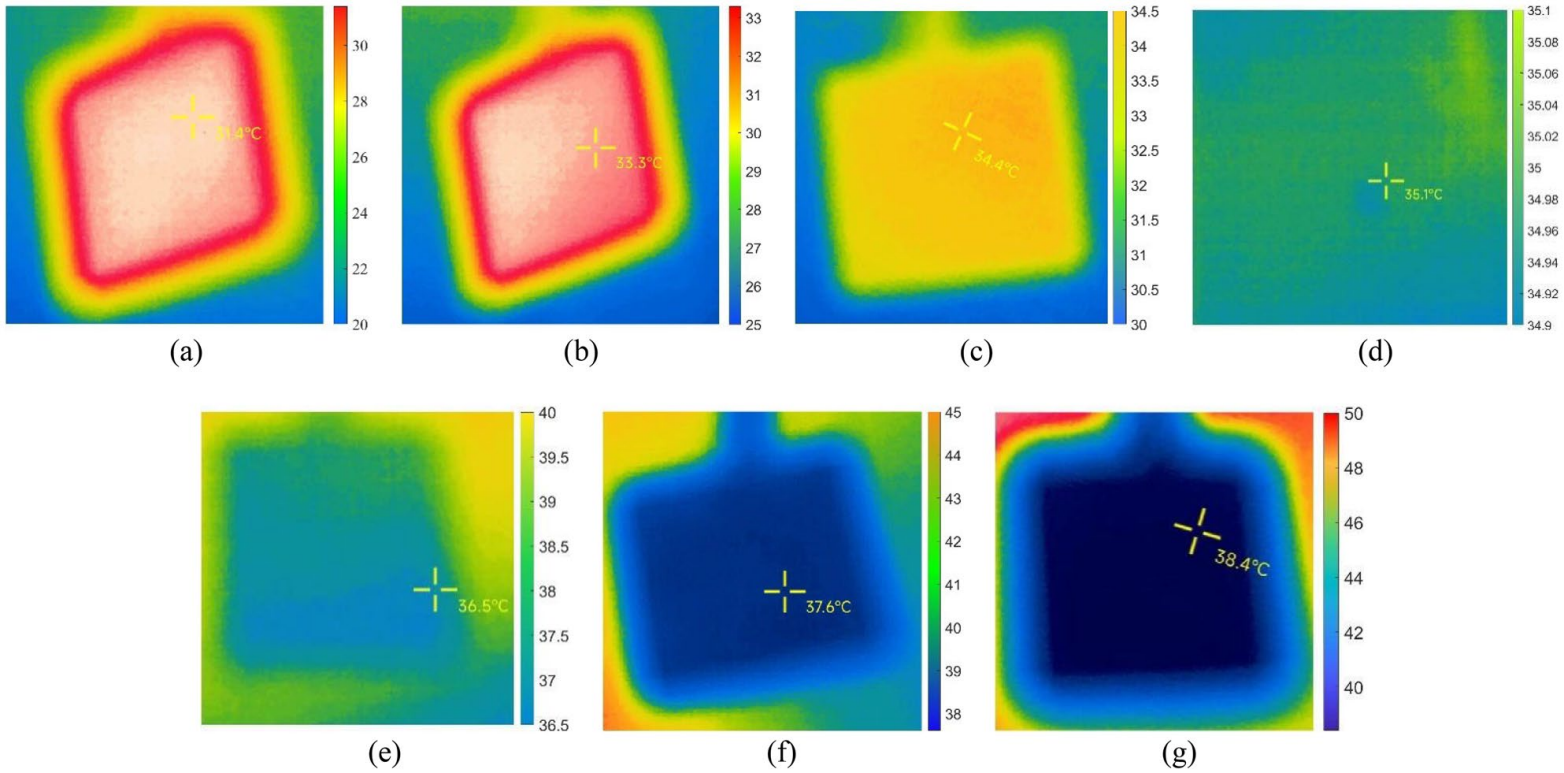
Images of the target taken in a normal environment with different environmental temperatures. The environmental temperatures for (**a**) to (**g**) are 20 °C, 25 °C, 30 °C, 35 °C, 40 °C, 45 °C, and 50 °C respectively.

During temperature measurements of the target using infrared thermal imaging, to ensure data accuracy, three sets of data were collected for the target in varying environmental temperatures. These measurements were conducted in a normal environment, a fog environment, and a smoke environment, respectively (Supplementary Figs. 1–10). The data for the target temperatures in the normal environment are detailed in Table 3. Additionally, Fig. 6 displays images from one of the groups of targets captured during the experiment.

**Table 3:** Target temperatures measured by infrared thermal imaging in normal environment with different environmental temperatures.

Simulate indoor environmental temperature	Actual temperature of the target object	Actual measured temperature of the target object			
		1	2	3	Mean
20 °C	35 °C	31.4 °C	32.0 °C	31.9 °C	31.77 °C
25 °C	35 °C	33.3 °C	33.5 °C	33.4 °C	33.40 °C
30 °C	35 °C	34.4 °C	34.8 °C	34.2 °C	34.47 °C
35 °C	35 °C	35.1 °C	35.4 °C	35.2 °C	35.23 °C
40 °C	35 °C	36.5 °C	36.7 °C	36.4 °C	36.53 °C
45 °C	35 °C	37.6 °C	37.4 °C	37.6 °C	37.53 °C
50 °C	35 °C	38.4 °C	38.6 °C	38.7 °C	38.57 °C

**Figure 7:**
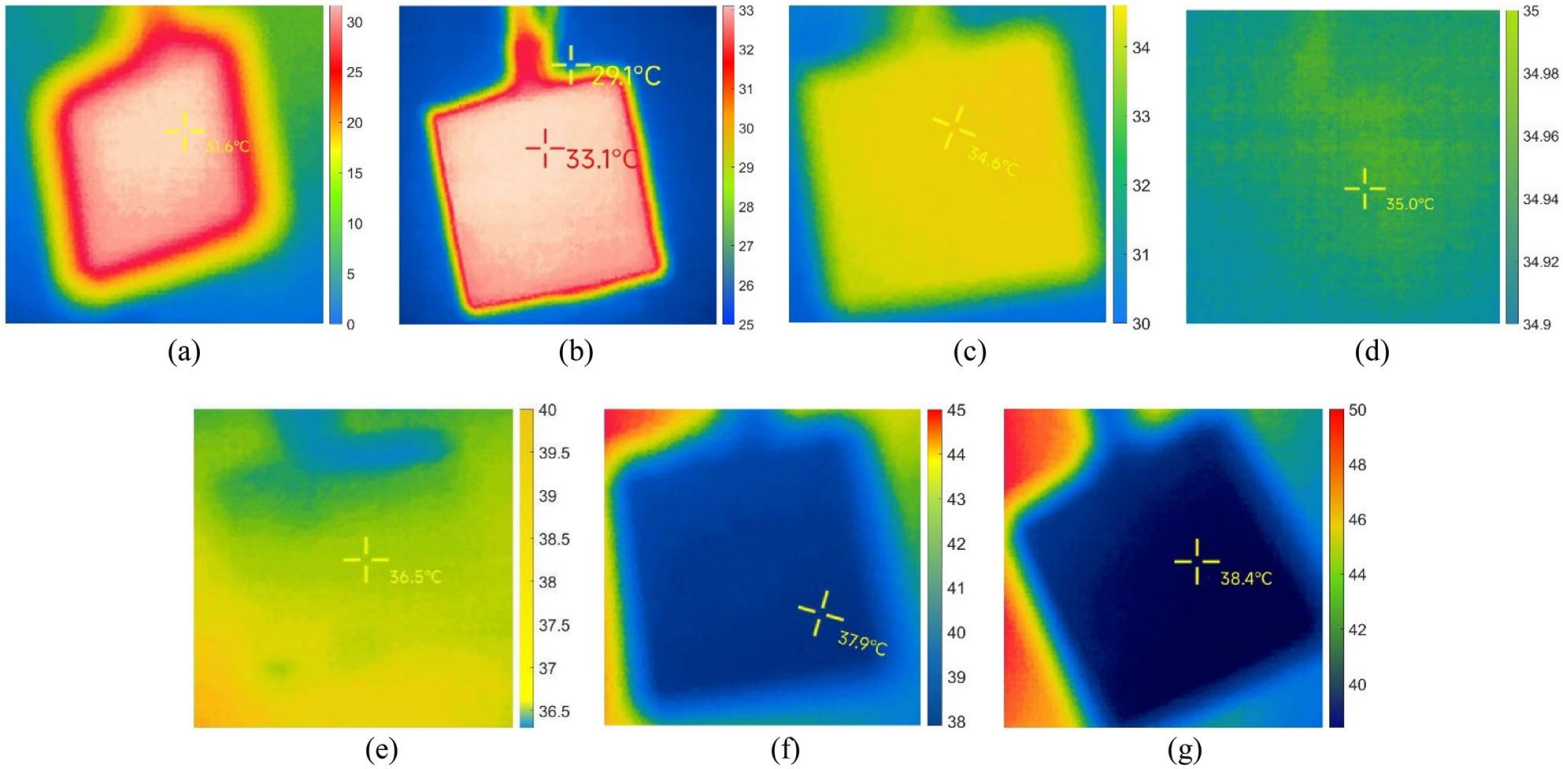
Images of the target taken in a smoke environment at different environmental temperatures. The environmental temperatures for (**a**) to (**g**) are 20 °C, 25 °C, 30 °C, 35 °C, 40 °C, 45 °C, and 50 °C respectively.

The temperature data of the targets measured in the smoke environment is presented in Table 4, while Fig. 7 showcases images from one of the sets of targets captured during the experiment.

**Table 4:** Target temperature data measured by infrared thermal imaging in smoke environment with different environmental temperatures.

Simulate indoor environmental temperature	Actual temperature of the target object	Actual measured temperature of the target object			
		1	2	3	Mean
20 °C	35 °C	31.6 °C	31.7 °C	32.2 °C	31.83 °C
25 °C	35 °C	33.1 °C	33.4 °C	33.5 °C	33.33 °C
30 °C	35 °C	34.6 °C	34.3 °C	34.0 °C	34.30 °C
35 °C	35 °C	35.0 °C	35.6 °C	35.2 °C	35.27 °C
40 °C	35 °C	36.5 °C	36.4 °C	36.5 °C	36.47 °C
45 °C	35 °C	37.9 °C	37.7 °C	37.5 °C	37.70 °C
50 °C	35 °C	38.4 °C	39.0 °C	38.9 °C	38.77 °C

**Figure 8:**
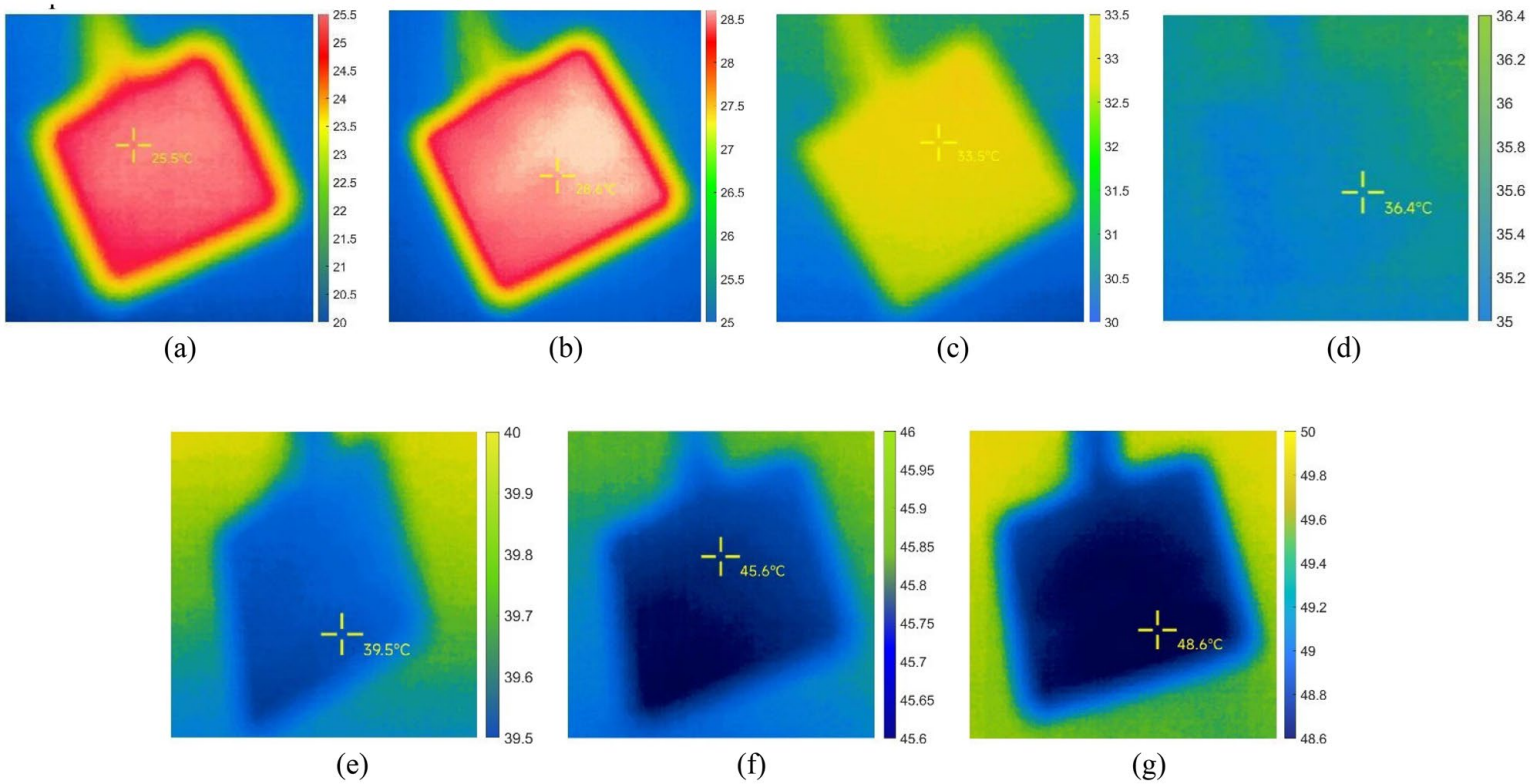
Images of the target taken in a foggy environment with different environmental temperatures. The environmental temperatures for (**a**) to (**g**) are 20 °C, 25 °C, 30 °C, 35 °C, 40 °C, 45 °C, and 50 °C respectively.

The temperature data of the targets measured in the foggy environment are shown in Table 5, and the images taken of one of the sets of targets are shown in Fig. 8.

**Table 5:** Target temperatures measured by infrared thermal imaging in foggy environment with different environmental temperatures.

Simulate indoor environmental temperature	Actual temperature of the target object	Actual measured temperature of the target object			
		1	2	3	Mean
20 °C	35 °C	25.5 °C	25.4 °C	25.7 °C	25.53 °C
25 °C	35 °C	28.6 °C	28.3 °C	28.6 °C	28.50 °C
30 °C	35 °C	33.5 °C	34.0 °C	33.6 °C	33.70 °C
35 °C	35 °C	36.4 °C	36.8 °C	36.0 °C	36.40 °C
40 °C	35 °C	39.5 °C	39.6 °C	39.3 °C	39.47 °C
45 °C	35 °C	45.6 °C	45.5 °C	44.9 °C	45.33 °C
50 °C	35 °C	48.6 °C	49.0 °C	49.1 °C	48.90 °C

### The analysis of experimental data

The visibility of smoke or fog can be calculated by formula (5), and the results of the visibility calculation of smoke are shown in Table 6 and the results of the visibility calculation of fog are shown in Table 7.

**Table 6:** The calculated data table for smoke visibility.

Environmental temperature	20 °C	25 °C	30 °C	35 °C	40 °C	45 °C	50 °C
Measuring distance	0.3m	0.3m	0.3m	0.3m	0.3m	0.3m	0.3m
Visibility	0.185m	0.185m	0.185m	0.185m	0.185m	0.185m	0.185m

**Table 7:** The calculated data table for fog visibility.

Environmental temperature	20 °C	25 °C	30 °C	35 °C	40 °C	45 °C	50 °C
Measuring distance	0.3m	0.3m	0.3m	0.3m	0.3m	0.3m	0.3m
Visibility	0.204m	0.204m	0.204m	0.204m	0.204m	0.204m	0.204m

**Figure 9:**
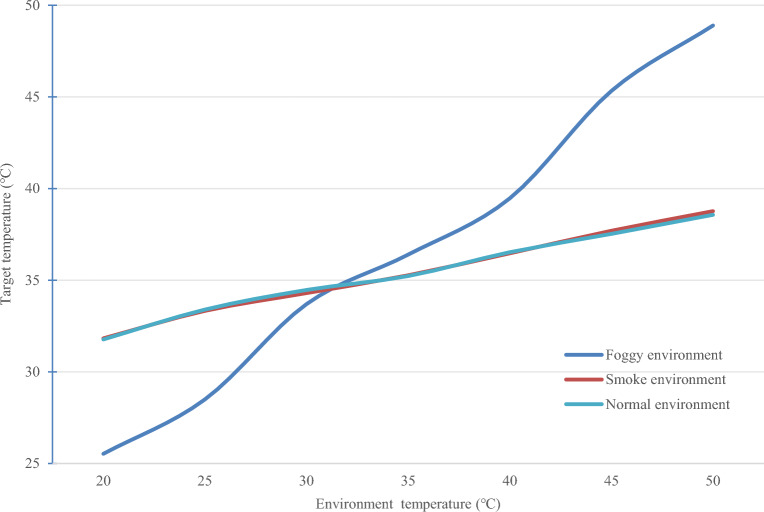
The line graphs fitted to the mean temperature of the targets.

The line graph of the measured infrared thermal imaging fitted to the average of the target temperatures in normal, smoke and fog environments at different environmental temperatures are shown in Fig. 9.

The optical system of the infrared thermal imager receives three types of effective radiation: target's own radiation, ambient reflected radiation, and atmospheric radiation^35^. As shown in Fig. 9, the measured target temperature by the infrared thermal imager increases with the rise of ambient temperature. In a normal environment, when the ambient temperature is 20 °C, the measured target temperature is 31.77 °C; at 50 °C ambient temperature, the measured target temperature rises to 38.57 °C. Additionally, the results indicate that with every 5 °C increase in ambient temperature, the measured target temperature increases by approximately 1 °C, showing a relatively gradual pace of growth. In a smoky environment, at 20 °C ambient temperature, the measured target temperature is 31.83 °C, and at 50 °C, it rises to 38.77 °C, with a similar trend of approximately 1 °C increase for every 5 °C rise in ambient temperature. However, in a foggy environment, the measured target temperature is notably different: at 20 °C ambient temperature, it's 25.53 °C, while at 50 °C, it reaches 48.90 °C. Moreover, the rate of increase in measured target temperature is stronger, ranging between 3 and 6 °C for every 5 °C rise in ambient temperature, highlighting a significant impact of foggy conditions on infrared thermal imaging measurements compared to the relatively minor impact in smoky environments.

Furthermore, across all environments, it's observed that when the ambient temperature ranges from 35 °C to 50 °C, the measured target temperature exceeds the actual value. Specifically, in foggy conditions, the measured target temperature is higher than in normal conditions, whereas in smoky conditions, it's relatively similar to normal conditions. Conversely, when the ambient temperature ranges from 20 °C to 35 °C, the measured target temperature is lower than the actual value, with again lower readings in foggy conditions compared to normal conditions, while measurements in smoky conditions remain comparable to those in normal conditions.

The influence of environmental temperature radiation causes temperature variations in fog droplets, sometimes exceeding ambient temperatures^36^. Consequently, significant errors in temperature measurement relative to actual values may occur. In foggy environments, where numerous fog droplets exist, measurement data errors for target objects are considerable. Conversely, in normal and smoky environments, where moisture molecules in the air are scarce, the errors in target object temperature measurements are relatively minor.

### The theoretical data

In a normal environment, as the target is measured at a close distance, ${\uptau }_{\text{a}}$ can be taken as an approximate value of 1. Substituting this into Eq. (6), the temperature measurement formula for an infrared thermal imaging system in a normal environment can be derived, as shown in Eq. (17).

17 $$ {\text{T}}_{{\text{r}}} { = }\left[ {{\varepsilon T}_{{0}}^{{\text{n}}} { + }\left( {{1} - {\upvarepsilon }} \right){\text{T}}_{{\text{u}}}^{{\text{n}}} } \right]^{{\text{1/n}}} $$

where $\upvarepsilon$ of the ceramic sheet is 0.8.

In the smoke environment, by formula (11), it can be calculated that the value of ${\uptau }_{\text{a}}$ in a foggy environment is 1.

In the fog environment, given that air humidity is essentially at 100%. By formula (7), it can be calculated that the value of ${\uptau }_{\text{a}}$ in a foggy environment is 0.2229. Substituting this into Eq. (6), the temperature measurement formula for infrared thermal imaging in the fog environment can be obtained, as shown in Eq. (18).

18 $$    {\text{T}}_{{\text{r}}} {\text{ = }}\left[ {\upvarepsilon \uptau _{{\text{a}}} {\text{T}}_{{\text{0}}}^{{\text{n}}} {\text{ + }}\left( {{\text{1}} - \upvarepsilon } \right)\uptau _{{\text{a}}} {\text{T}}_{{\text{u}}}^{{\text{n}}} {\text{ + }}\left( {{\text{1}} - \uptau _{{\text{a}}} } \right){\text{T}}_{{\text{a}}}^{{\text{n}}} } \right]^{{{\text{1/n}}}}    $$

where $\upvarepsilon$ of the ceramic sheet is 0.8.

In a normal environment and a smoke environment, the temperature data of the target measured by thermal imaging under theoretical conditions can be calculated using formula (17). For the fog environment, the temperature data of the target measured by thermal imaging under theoretical conditions can be calculated using formula (18). The calculated temperature data of the target are detailed in Table 8.

**Table 8:** Calculated temperatures of targets in each environment.

Simulate indoor environmental temperature	Actual temperature of the target object	Calculate temperature in normal environment	Calculate temperature in smoke environment	Calculate temperature in foggy environment
20 °C	35 °C	32.17 °C	32.17 °C	22.85 °C
25 °C	35 °C	33.08 °C	33.08 °C	26.86 °C
30 °C	35 °C	34.17 °C	34.17 °C	30.91 °C
35 °C	35 °C	35.00 °C	35.00 °C	35.00 °C
40 °C	35 °C	36.02 °C	36.02 °C	39.13 °C
45 °C	35 °C	37.08 °C	37.08 °C	43.29 °C
50 °C	35 °C	38.18 °C	38.18 °C	47.48 °C

**Figure 10:**
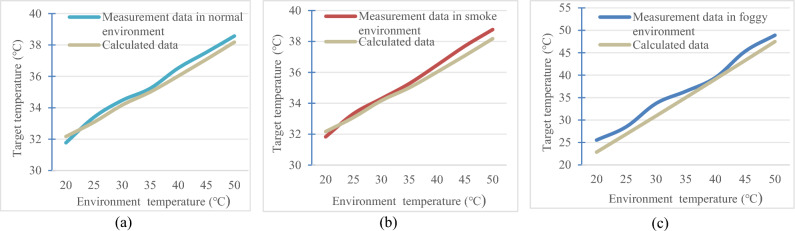
Measured temperatures of the target object compared to theoretical temperatures in different environments. (**a**) Normal environment. (**b**) Smoky environment. (**c**) Foggy environment.

The calculated data in the normal environment, along with the actual measured target data fitted by the line graph shown in Fig. 10a, are presented. Similarly, the calculated data in the smoke environment, along with the actual measured target data fitted by the line graph shown in Fig. 10b, are provided. The line graph of the calculated data in the fog environment fitted to the actual measured target data is shown in Fig. 10c.

From Fig. 10, it can be observed that the target's temperature measured in the experiment is generally consistent with the theoretical temperature of the target. This result indicates that the measurement method and instruments used in the experiment possess good accuracy and reliability. Additionally, from the theoretical data, it is evident that foggy conditions have a significant impact on infrared thermal imaging, while smoky environments have a relatively minor effect on infrared thermal imaging.

## Conclusion

This paper delves into the impact of smoke or fog on infrared radiation at different ambient temperatures. Although we have not measured the impact of environments with different concentrations of smoke or fog on infrared radiation, we have also drawn the following conclusions:

Due to the influence of ambient temperature radiation causing temperature changes in fog droplets, while smoke particles are less affected by this phenomenon, in normal, smoky, and foggy environments, when the ambient temperature is lower than the target object's temperature, the measured target temperature by infrared thermal imaging is lower than the actual temperature. Conversely, when the ambient temperature is higher than the target temperature, the measured target temperature by infrared thermal imaging is higher than the actual temperature. Moreover, infrared thermal imaging is significantly affected in foggy environments, while its impact is minor in smoky environments and even negligible.

In the future, we will refine our experimental design to comprehensively study the effects of environments with varying concentrations of smoke or fog on infrared radiation.

### Supplementary Information

Supplementary Figures.

## Data Availability

Data is provided within the supplementary information files.
