# Supplementary material for: The research on infrared radiation affected by smoke or fog in different environmental temperatures
Source: Sci Rep. 2024 Jun 22;14:14410. doi: 10.1038/s41598-024-65462-x (PMC11193785; doi:10.1038/s41598-024-65462-x)
Supplement: Supplementary file 1 — Supplementary Figures. [file 41598_2024_65462_MOESM1_ESM.docx]

**Infrared thermal imaging measurement experimental data**

| 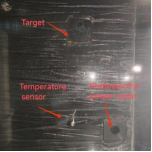 | 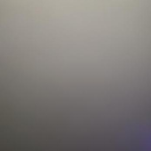 | 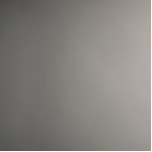 |
| --- | --- | --- |
| (a) | (b) | (c) |

**Figure 1.**  Images taken by the visible camera in normal, smoke and foggy environments. (a) Normal environment. (b) Smoke environment. (c) In a foggy environment.

| 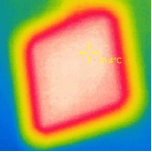 | 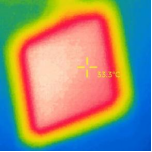 | 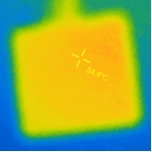 | 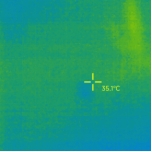 |
| --- | --- | --- | --- |
| (a) | (b) | (c) | (d) |
| 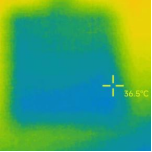 | 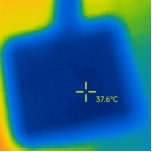 | 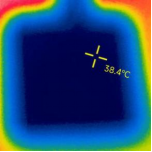 |  |
| (e) | (f) | (g) |  |

**Figure 2.** The first set of target images taken under normal environment with different environment temperatures. (a) Environmental temperature20℃. (b) Environmental temperature25℃. (c) Environmental temperature30℃. (d) Environmental temperature35℃. (e) Environmental temperature40℃. (f) Environmental temperature45℃. (g) Environmental temperature50℃.

| 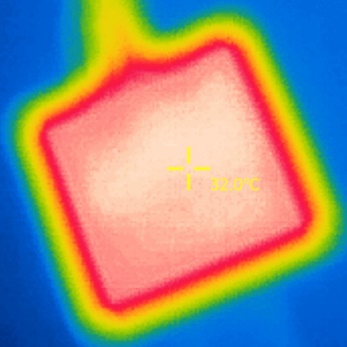 | 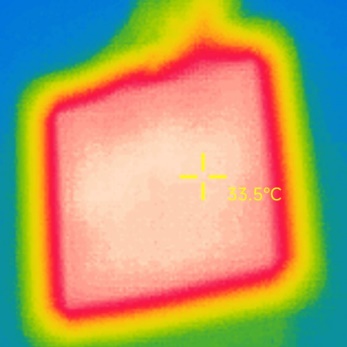 | 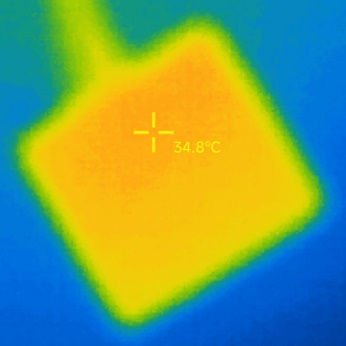 | 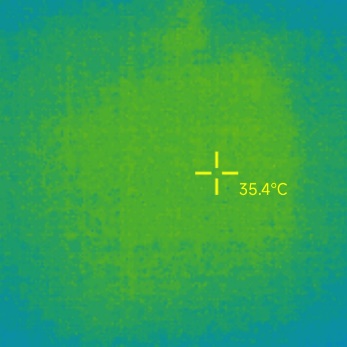 |
| --- | --- | --- | --- |
| (a) | (b) | (c) | (d) |
| 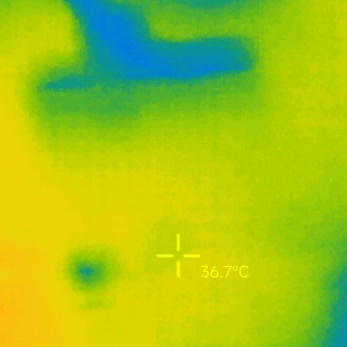 | 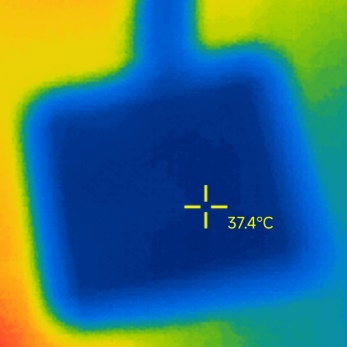 | 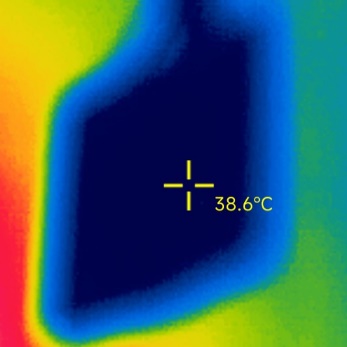 |  |
| (e) | (f) | (g) |  |

**Figure 3.** The second set of target images taken under normal environment with different environment temperatures. (a) Environmental temperature20℃. (b) Environmental temperature25℃. (c) Environmental temperature30℃. (d) Environmental temperature35℃. (e) Environmental temperature40℃. (f) Environmental temperature45℃. (g) Environmental temperature50℃.

| 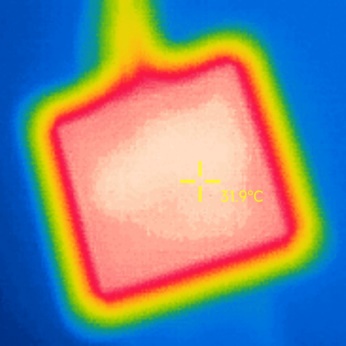 | 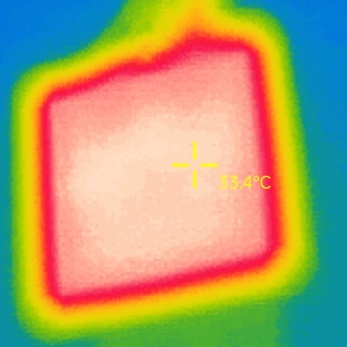 | 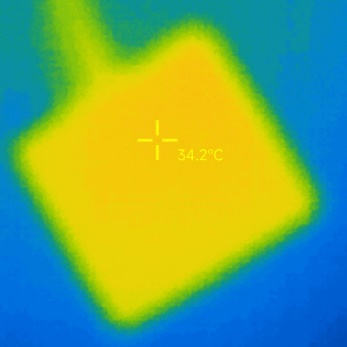 | 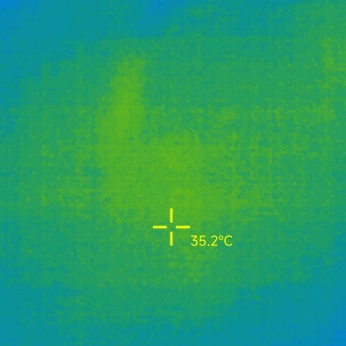 |
| --- | --- | --- | --- |
| (a) | (b) | (c) | (d) |
| 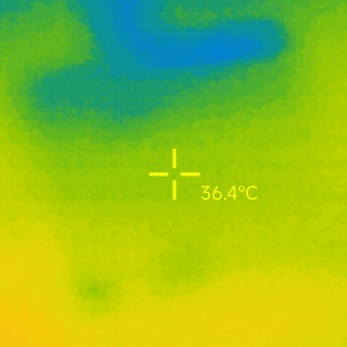 | 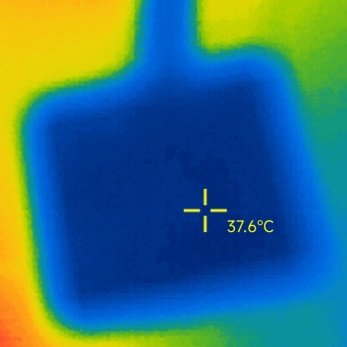 | 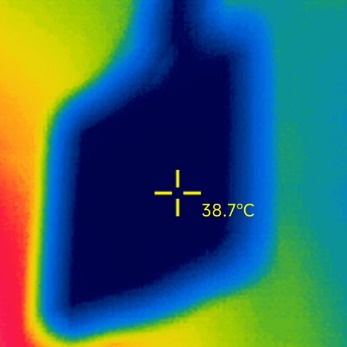 |  |
| (e) | (f) | (g) |  |

**Figure 4.** The third set of target images taken under normal environment with different environment temperatures. (a) Environmental temperature20℃. (b) Environmental temperature25℃. (c) Environmental temperature30℃. (d) Environmental temperature35℃. (e) Environmental temperature40℃. (f) Environmental temperature45℃. (g) Environmental temperature50℃.

| 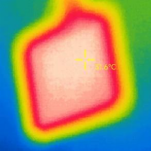 | 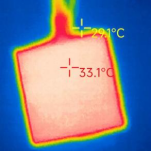 | 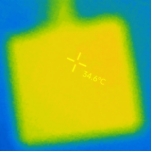 | 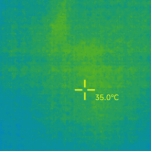 |
| --- | --- | --- | --- |
| (a) | (b) | (c) | (d) |
| 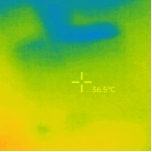 | 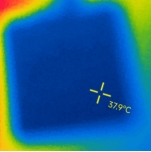 | 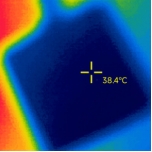 |  |
| (e) | (f) | (g) |  |

**Figure 5.** The first set of target images taken under smoke environment with different environment temperatures. (a) Environmental temperature20℃. (b) Environmental temperature25℃. (c) Environmental temperature30℃. (d) Environmental temperature35℃. (e) Environmental temperature40℃. (f) Environmental temperature45℃. (g) Environmental temperature50℃.

| 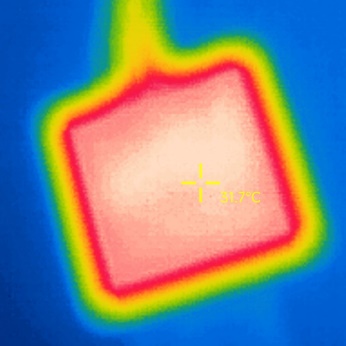 | 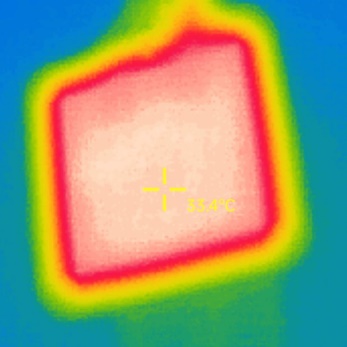 | 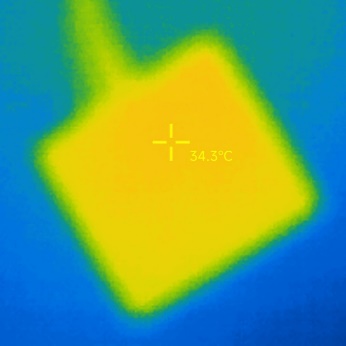 | 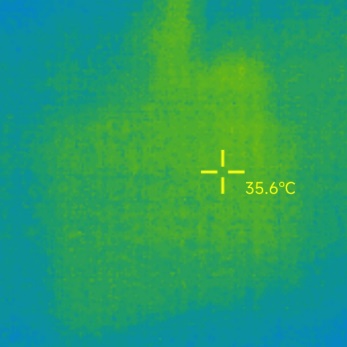 |
| --- | --- | --- | --- |
| (a) | (b) | (c) | (d) |
| 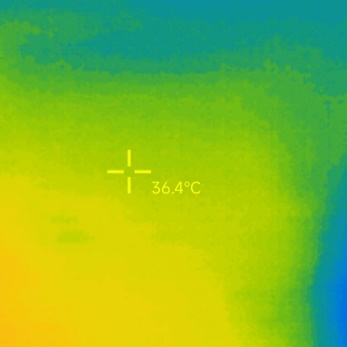 | 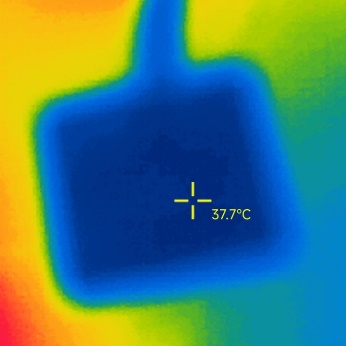 | 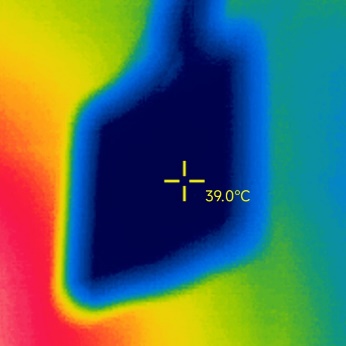 |  |
| (e) | (f) | (g) |  |

**Figure 6.** The second set of target images taken under smoke environment with different environment temperatures. (a) Environmental temperature20℃. (b) Environmental temperature25℃. (c) Environmental temperature30℃. (d) Environmental temperature35℃. (e) Environmental temperature40℃. (f) Environmental temperature45℃. (g) Environmental temperature50℃.

| 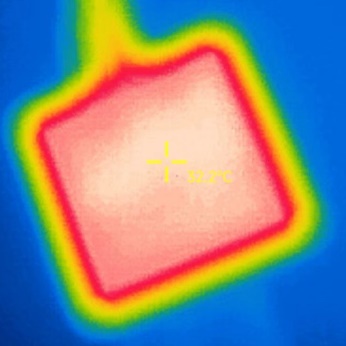 | 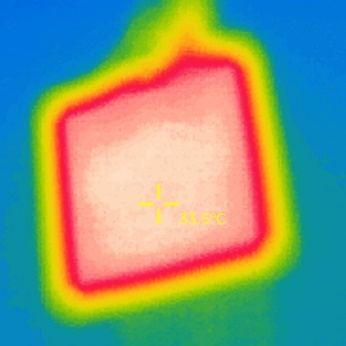 | 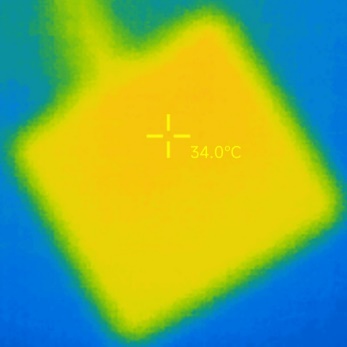 | 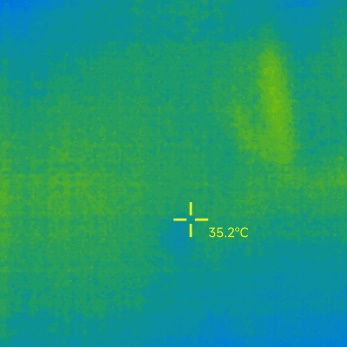 |
| --- | --- | --- | --- |
| (a) | (b) | (c) | (d) |
| 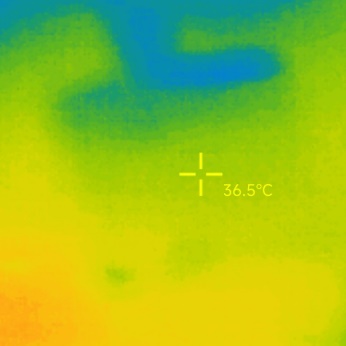 | 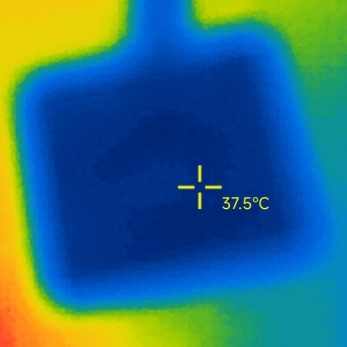 | 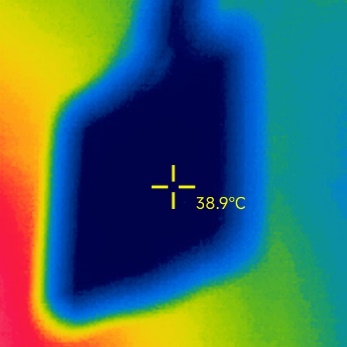 |  |
| (e) | (f) | (g) |  |

**Figure 7.** The third set of target images taken under smoke environment with different environment temperatures. (a) Environmental temperature20℃. (b) Environmental temperature25℃. (c) Environmental temperature30℃. (d) Environmental temperature35℃. (e) Environmental temperature40℃. (f) Environmental temperature45℃. (g) Environmental temperature50℃.

SS

| 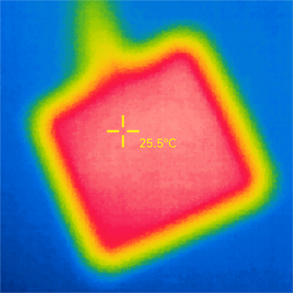 | 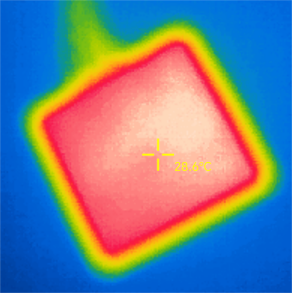 | 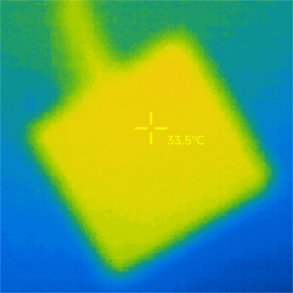 | 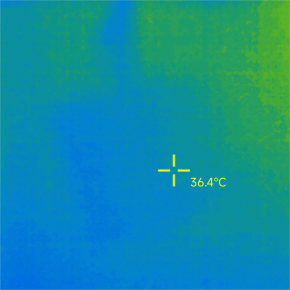 |
| --- | --- | --- | --- |
| (a) | (b) | (c) | (d) |
| 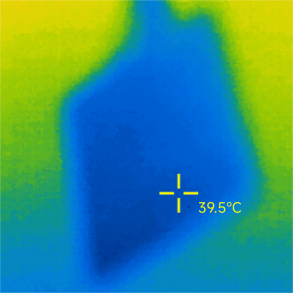 | 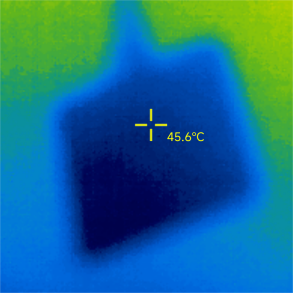 | 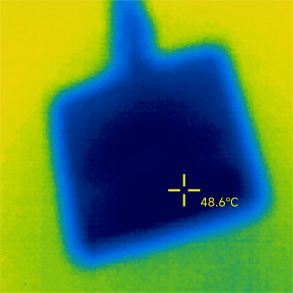 |  |
| (e) | (f) | (g) |  |

**Figure 8.** The first set of target images taken under foggy environment with different environment temperatures. (a) Environmental temperature20℃. (b) Environmental temperature25℃. (c) Environmental temperature30℃. (d) Environmental temperature35℃. (e) Environmental temperature40℃. (f) Environmental temperature45℃. (g) Environmental temperature50℃.

| 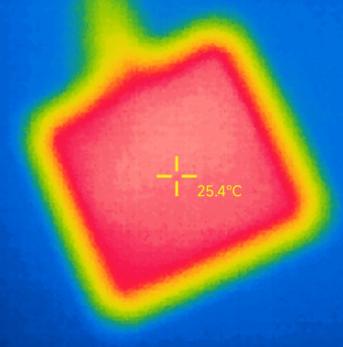 | 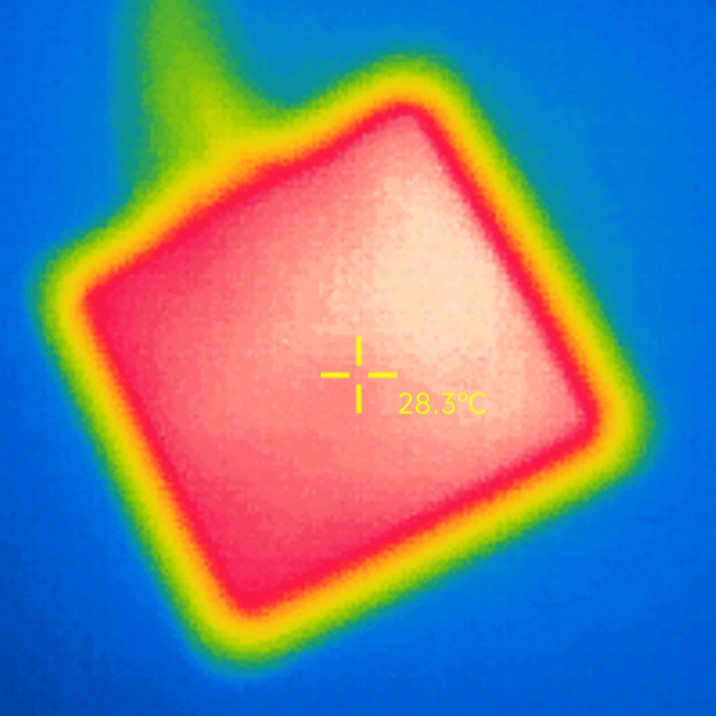 | 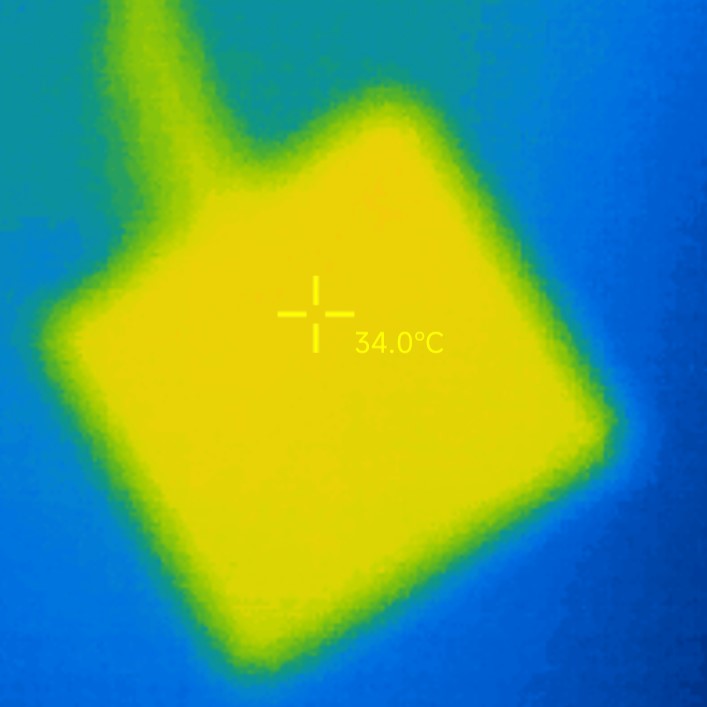 | 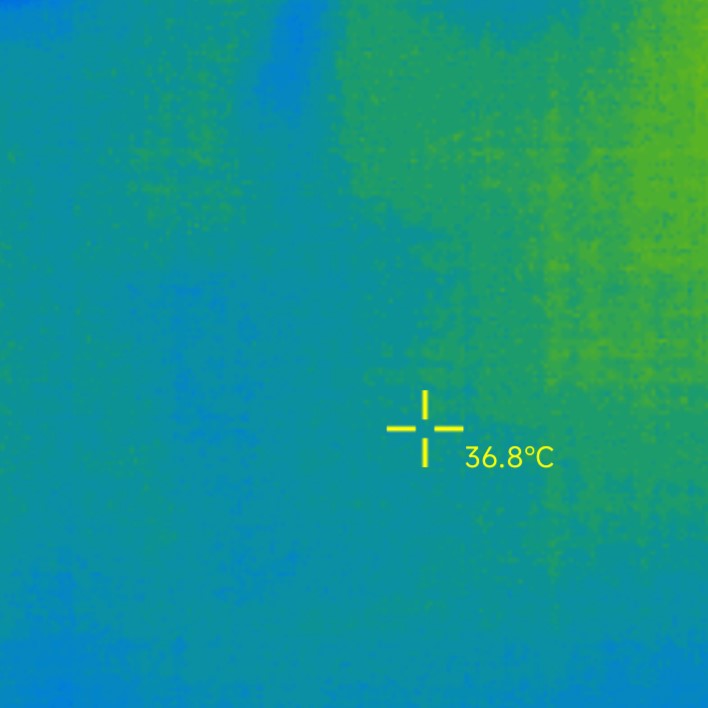 |
| --- | --- | --- | --- |
| (a) | (b) | (c) | (d) |
| 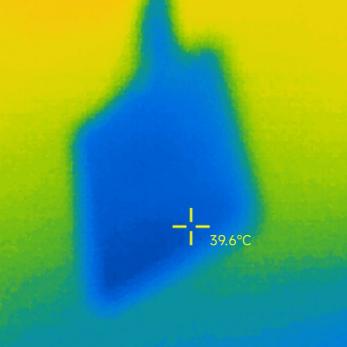 | 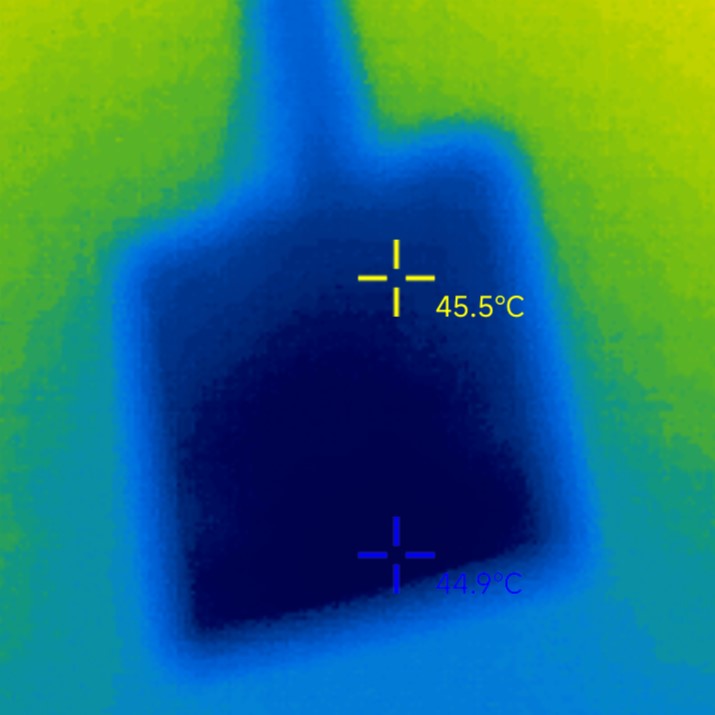 | 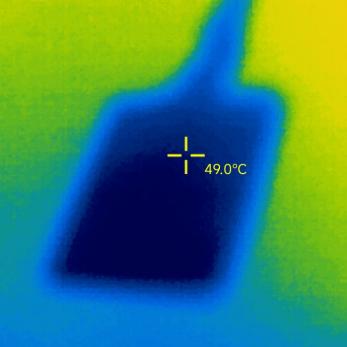 |  |
| (e) | (f) | (g) |  |

**Figure 9.** The second set of target images taken under foggy environment with different environment temperatures. (a) Environmental temperature20℃. (b) Environmental temperature25℃. (c) Environmental temperature30℃. (d) Environmental temperature35℃. (e) Environmental temperature40℃. (f) Environmental temperature45℃. (g) Environmental temperature50℃.

| 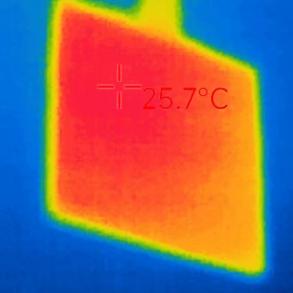 | 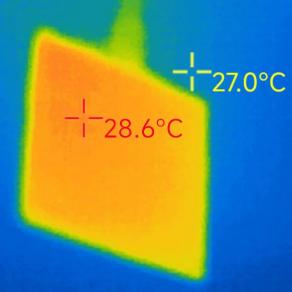 | 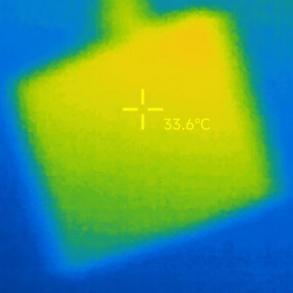 | 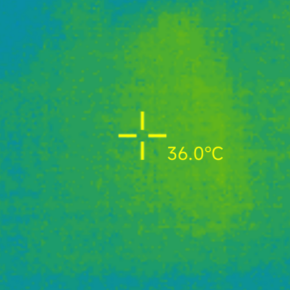 |
| --- | --- | --- | --- |
| (a) | (b) | (c) | (d) |
| 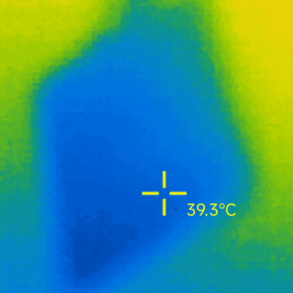 | 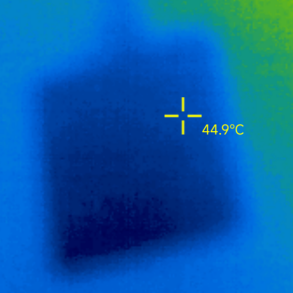 | 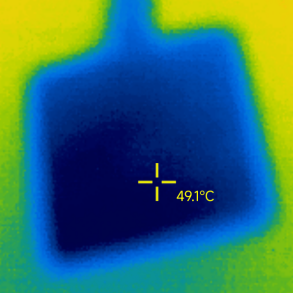 |  |
| (e) | (f) | (g) |  |

**Figure 10.** The third set of target images taken under foggy environment with different environment temperatures. (a) Environmental temperature20℃. (b) Environmental temperature25℃. (c) Environmental temperature30℃. (d) Environmental temperature35℃. (e) Environmental temperature40℃. (f) Environmental temperature45℃. (g) Environmental temperature50℃.
